# Scalp Reconstruction With Local Flaps: A Case Series

**DOI:** 10.7759/cureus.91366

**Published:** 2025-08-31

**Authors:** Yosira G López-Alvarado, Jose A Acosta-Flores, Gabriel García-González, Mauricio M García-Pérez, Everardo Valdés-Flores, Luis Carlos Lozano-Carrillo

**Affiliations:** 1 General Surgery, Instituto de Seguridad y Servicios Sociales de los Trabajadores del Estado (ISSSTE), Monterrey, MEX; 2 Plastic, Aesthetic and Reconstructive Surgery, “Dr. José Eleuterio González” University Hospital, Monterrey, MEX; 3 General Surgery, Hospital Metropolitano, San Nicolás de los Garza, MEX; 4 Anatomy, “Dr. José Eleuterio González” University Hospital, Monterrey, MEX

**Keywords:** flap, local flap, reconstruction, scalp, surgery

## Abstract

The scalp, a crucial protective barrier for intracranial structures, undergoes intricate reconstruction procedures, demanding detailed anatomical knowledge. The mnemonic SCALP identifies its layers, influencing successful aesthetic reconstruction. Vascular considerations, orchestrated by branches from carotid arteries, sustain scalp health. The multifaceted nature of scalp defects, driven by diverse causes, underscores reconstruction complexity. Effective surgical planning, assessing intrinsic and extrinsic factors, is imperative. Insights from the Plastic and Reconstructive Surgery Department at the “Dr. José Eleuterio González” University Hospital, particularly on local flaps, are shared, highlighting their significance in achieving optimal outcomes. All patients treated during this period were included, with flap types chosen based on defect size for tension-free coverage. Despite the depth of the affected site, local flap reconstruction was successfully employed. The study details flap nourishment, defect location descriptions, and surgical planning, meticulously assessing intrinsic and extrinsic factors. Eighteen patients underwent successful scalp reconstruction, primarily for traumatic (55.5%) and oncological (44.4%) reasons. Male patients dominated (72.2%), and interventions varied across regions, with the parietal area being the most common (44.4%). The postoperative period showed a lack of complications, with all flaps surviving and no wound dehiscence, affirming the efficacy of the reconstruction procedures. The findings contribute valuable insights into optimizing scalp reconstruction outcomes.

## Introduction

The scalp, a highly specialized tissue, serves as a vital physical, immunological, and thermal insulation barrier safeguarding intracranial structures. The mnemonic SCALP (skin, subcutaneous tissue, galea aponeurotica, loose areolar tissue, and pericranium) is frequently used to refer to the layers that make up the architecture of the scalp, each of which has unique characteristics that favor a successful aesthetic reconstruction. Detailed anatomical knowledge is critical to the planning of scalp reconstruction procedures.

These anatomical considerations play a pivotal role in successfully evaluating a well-vascularized flap. The superficial aponeurotic galea, the blood, nerve, and lymphatic vessels are strategically positioned, highlighting their relevance in the flap dissection process. Deeper within this anatomy lies a layer of loose connective tissue, facilitating optimal mobility of the overlying tissue and simplifying the dissection procedure. The easily dissected layer is routinely included in flap dissection, contributing to the overall efficacy of the procedure. Ultimately, the deepest layer, the pericranium, provides irrigation to the skull bone and serves as a vascularized surface for graft applications, aligning with the specific reconstruction plan [1,2].

Scalp irrigation is orchestrated by branches stemming from both the internal carotid artery and the external carotid artery as well; the anterior aspect receives vascular supply through the supratrochlear and supraorbital arteries, contributing to the vitality of the frontal region. Simultaneously, the lateral portion benefits from the superficial temporal and posterior auricular arteries, ensuring proper irrigation to this area. The posterior region is adeptly supplied by the occipital artery, completing the comprehensive network of vascular support crucial for scalp health and surgical procedures [1].

The multifaceted nature of scalp defects, stemming from diverse etiologies such as trauma, neoplastic or benign lesions, burns, radiation, infections, congenital abnormalities, and aesthetic concerns, and its particularities, such as restricted expandability of the galea and the lack of tissue in the area, underscores the complexity of reconstruction [1,3-5].

Effective surgical planning necessitates a comprehensive evaluation of intrinsic and extrinsic factors. Surgeons must meticulously assess patients' health information, encompassing chronic diseases, smoking habits, corticoid use, radiation history, social situation, and commitment to postoperative care [1].

This article shares insights gleaned from the clinical experience of the Plastic and Reconstructive Surgery Department at the “Dr. José Eleuterio González” University Hospital in the area of scalp reconstruction [6]. An in-depth discussion of various techniques is provided, with a particular emphasis on local flaps, recognized as the method of choice for achieving optimal outcomes.

This study aims to present our department’s experience in scalp reconstruction using local flaps, describing patient demographics, defect characteristics, flap selection, surgical technique, and short-term outcomes.

## Materials and methods

This retrospective observational study included all patients who underwent scalp reconstruction procedures using local flaps, treated by the Plastic and Reconstructive Surgery Department at the “Dr. José Eleuterio González” University Hospital between March 2019 and June 2022.

Clinical, surgical, and demographic data were retrospectively collected. Surgical indications, anatomical defect location, type of flap used, defect size, and arteries responsible for flap perfusion were documented. Measurements were obtained in two stages: first, the lesion size was recorded prior to resection, and second, following debridement. Flap dimensions were planned based on the post-debridement measurements to ensure adequate coverage.

The types of local flaps employed included double rotation, transposition, advancement, and triple rotation flaps. The selection of flap type was based on the size, shape, and location of the defect, prioritizing complete, tension-free coverage. Defect depth was not considered a contraindication for the use of local flaps in any case.

The anatomical location of the defects was classified according to the predominantly affected scalp region: frontal, parietal, temporal, and parieto-occipital. In all cases, superficial arterial vessels such as the superficial temporal artery and occipital artery were preserved and utilized to ensure adequate flap perfusion.

Immediate and outpatient postoperative follow-up was performed to assess flap viability, the presence of complications (such as wound dehiscence, necrosis, or infection), and the aesthetic and functional evolution of each case. Postoperative management included pain control and antibiotic prophylaxis. Patients were monitored continuously during a two-day inpatient stay, with flap perfusion assessed via capillary refill and temperature checks every two hours for the first 12 hours, then every four hours for the following 36 hours until discharge.

Follow-up

All patients were scheduled for weekly outpatient follow-up after discharge, with suture removal performed between two and three weeks, depending on the case.

## Results

A total of 18 patients underwent scalp reconstruction using local flaps during the study period. Of these, 13 (72.2%) were male and five (27.7%) female, with a mean age of 59.6 years. The most frequent etiologies were traumatic injuries (55.5%), predominantly electrical burns (33.3%), followed by oncologic causes (44.4%).

Defects were located in the parietal (44.4%), frontoparietal (22.2%), parietooccipital (22.2%), occipital (5.5%), and a single complex case involving the frontoparietotemporal and zygomaticomaxillary region (5.5%). Defect sizes ranged from 8 cm² to 112 cm², all successfully managed with local flap techniques.

The most commonly used techniques included double rotation flaps (n=7), transposition flaps (n=6), advancement flap (n=1), and triple rotation flap (n=1). In three cases, local flap coverage was complemented with skin grafts due to extensive or full-thickness defects.

**Figure 1:**
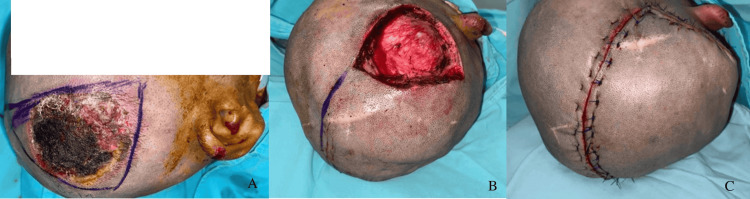
Electrical burn in the right parietal region. A: scalp area of 35 cm^2^ before debridement; B: complete surgical debridement of the lesion with periosteum exposure; C: immediate postoperative result of transposition flap to cover the defect.

**Figure 2:**
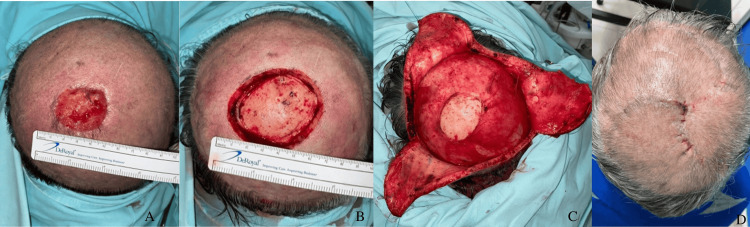
Basal cell carcinoma in the vertex. A: lesion before resection of 5 x 5 cm; B: defect of 36 cm^2^ after oncological resection; C: transoperative observation of rotational flap dissection before defect closure; D: postoperative outcome at follow-up with favourable evolution.

**Figure 3:**
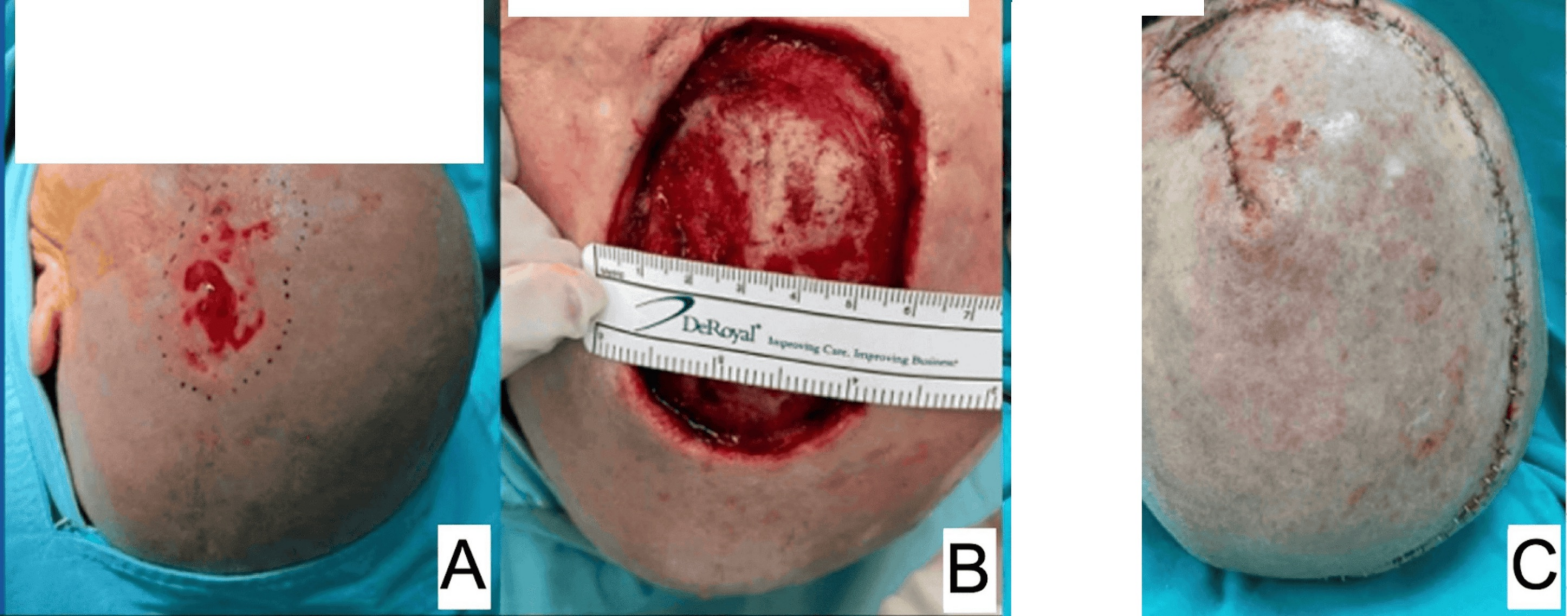
Squamous cell carcinoma in the left frontoparietal region. A lesion of 6 x 4 cm in our patient. A: lesion in the trans surgical area after debridement, demarcating the resection site; B: defect of 48 cm^2^ after oncological resection; C: immediate postoperative result of transposition flap for defect coverage.

**Figure 4:**
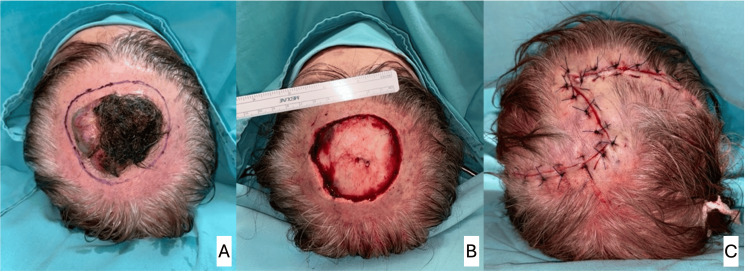
Papillary syringocystadenoma in the vertex. A: lesion measuring 6 x 5 cm before resection; B: defect of 49 cm^2^ after oncological resection; C: immediate postoperative result with defect closure with double O-Z rotational flap.

**Figure 5:**
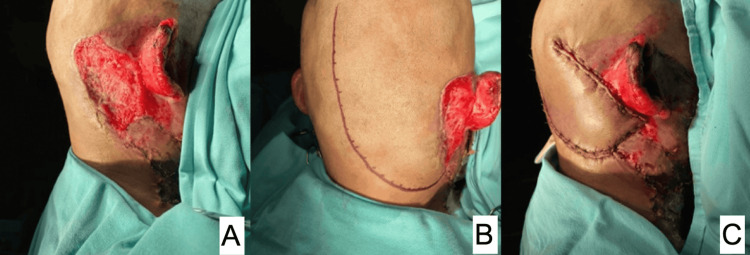
Electrical burn in the right temporal region. A: lesion measuring 8 x 6 cm with granulation tissue in the right retroarticular position; B: flap marking before mobilization; C: immediate postoperative result of defect coverage with transposition flap.

All patients were evaluated in the immediate postoperative period and received outpatient follow-up. Flap viability, presence of complications (e.g., dehiscence, necrosis, infection), and aesthetic/functional outcomes were assessed. All cases showed complete flap survival and favorable healing outcomes, illustrated in Figures 1-5. A comprehensive summary of patient data, including etiology, location, wound size, flap dimensions, depth, and arterial irrigation, is presented in Table 1.

**Table 1:** Characteristics of the patients included in the study. Distribution by sex, age, etiology, anatomical location of the defect, defect and flap size, depth of the lesion, type of flap used, and vascular supply employed for scalp reconstruction.

N.	Sex	Age	Etiology	Location	Wound size (cm^2^)	Flap size (cm^2^)	Deepness	Flap type/irrigation
1	M	59	Electrical burn	Frontoparietal	90	200/ 190	No periosteum	Double rotation / superficial temporal artery
2	M	24	Electrical burn (Figure 1)	Frontoparietal	35	80 / 88	No skin nor galea	Double rotation / superficial temporal artery
3	M	23	Avulsion	Frontoparietal	32	56	No periosteum	Advancement / superficial temporal artery
4	M	39	Electrical burn	Parietal	35	140	No skin nor galea	Transposition / occipital artery
5	M	76	Basal cell carcinoma (Figure 2)	Parietal	36	130 / 142 / 136	No periosteum	Triple rotation / superficial and occipital temporal artery
6	M	81	Epidermoid carcinoma (Figure 3)	Frontoparietal	48	120	No periosteum	Transposition/ superficial and occipital temporal artery
7	F	72	Basal cell carcinoma	Parietooccipital	20	70	No periosteum	Transposition / occipital artery
8	F	6	Avulsion	Parietal	12	80	No skin nor galea	Transposition / occipital artery
9	F	72	Epidermoid carcinoma	Parietooccipital	45	92 / 80	No periosteum	Double rotation / superficial temporal artery
10	F	81	Syringocystadenoma papilliferum (Figure 4)	Parietal	49	99 / 90	No skin nor galea	Double rotation / superficial temporal artery
11	M	23	Avulsion (Figure 5)	Parietal	24	72	No skin nor galea	Double rotation / superficial temporal artery
12	M	38	Electrical burn	Temporal	56	96	No skin nor galea	Transposition / occipital artery
13	F	67	Basal cell carcinoma	Parietal	8	40 / 47	No periosteum	Double rotation / superficial temporal artery
14	M	31	Electrical burn	Parietooccipital	86	138	No periosteum	Transposition + skin graft / superficial temporal artery
15	M	56	Electrical burn	Parietooccipital	108	140 / 170	No periosteum	Double rotation / superficial temporal artery
16	M	86	Basal cell carcinoma	Frontoparietotemporal + zigomaticomaxillar	112	220	No skin	Transposition + skin graft/ suprathroclear
17	M	64	Basal cell carcinoma	Parietal	30	60 / 68	No skin	Double rotation / superficial temporal artery
18	M	68	Electrical burn	Parietal	45	134	No periosteum	Transposition + skin graft/ occipital artery

All patients achieved complete coverage of the defect with no flap loss, no wound dehiscence, and no postoperative complications.

## Discussion

The optimal reconstructive approach of the scalp must be individualized, with considerations for reconstruction objectives based on the size, type, and location of the defect. This tailoring ensures the selection of the most suitable course of action for each patient [6].

Trauma and oncology are the most predominant reasons for the need for scalp reconstruction [7]. Despite the drop in cases due to COVID-19 restriction measures in 2020 and 2021, trauma increased its incidence in 2022 and 2023 at the emergency departments in developed countries [8-13].

The scalp is a region naturally exposed to high amounts of ultraviolet rays, so the development of primary and metastatic cancer is frequent at this site, with basal cell carcinoma, squamous cell carcinoma, and melanoma predominating. The primary treatment of these tumors is surgical resection, so scalp reconstruction is a commonly performed procedure [2,12,13].

The utilization of the reconstructive compass stands as a pivotal strategy in addressing scalp defects. A nuanced assessment of complexity and procedural needs is essential; success in reconstruction hinges on a thorough consideration of the anatomic layers of the scalp [1]. In our study population, extensive debridement following injuries such as scalp avulsion secondary to car accidents and electrical burn injuries was also part of the procedures that required subsequent reconstruction with a local flap.

It is known that primary closure needs to be considered in cases of small defects (less than 3 cm) and when the patient's characteristics allow it [14,15]. The preferred and practical approach to address scalp defects lies in the strategic use of local flaps, particularly when managing defects smaller than 150 cm². Beyond providing optimal coverage, this method offers significant advantages in mitigating alopecia and proves valuable in challenging scenarios like infections, radiotherapy, necrosis, and aesthetic goals [3,16,17]. The employment of tissue expansion techniques further enhances the reconstructive repertoire, considering factors such as defect size, trauma, and tissue distortion [18].

Grafting is considered a viable alternative when the defect is classified as partial-thickness and 50% of the scalp is lost. Also, it is important to have an intact periosteum in the recipient bed, with the inconvenience of partial or total graft loss in patients receiving post-reconstruction radiation. Likewise, closure by second intention is an alternative that is rarely practiced due to the risk of complications, such as wound retraction, infection, ulceration, skull exposure, and necrosis [19].

A preferred alternative includes local advancement, transposition, and rotation flaps. Considering that a circular residual defect is a common scenario after the resection of a scalp lesion, the spiral rotation flap is one of the most suitable options for performing a successful reconstruction [19].

The use of pediculated flaps and free flaps is reserved for cases where there are multiple previous surgeries, previously failed reconstruction, infection, or reconstruction of an area of full thickness that is not possible to close with primary closure [19]. In multiple cases, a combination of advance and rotation flaps is needed to achieve proper closure of a defect in the scalp. A proper example is defects of 50 cm² in the vertex, where bilateral lateral advancement flaps combined with an occipital rotation flap work as a good alternative [20].

In the case of our population, the largest defect was 90 cm², so reconstruction was carried out by performing local flaps in all cases, this being the best alternative in patients who require a defect closure without the need to take tissue from a donor site. In addition, performing a local flap on the scalp for reconstruction avoids secondary alopecia and offers full coverage of the defect after an oncological resection to continue their radiation treatment if necessary.

As for aesthetic considerations, incisions should be made in areas of the scalp skin to minimize the visible scar. In addition, it is sought to respect the hair follicles at the time of the incision to avoid areas of alopecia in the residual scar. The local flap must be designed to respect the correct orientation and alignment of the hair follicles in the area to be reconstructed. Finally, to improve the aesthetic result, always consider a hair transplant [18].

This study has several limitations worth noting. As a retrospective analysis with a limited sample size, the generalizability of the findings is restricted. Furthermore, its single-center design limits the applicability of the results to broader clinical contexts. Lastly, the absence of long-term follow-up precludes assessment of functional and aesthetic outcomes over time.

## Conclusions

Several surgical techniques, including primary closure, pedicled or free flaps, and skin grafts, have demonstrated success in repairing scalp defects. Notably, scalp tissue emerges as the most suitable replacement, emphasizing the significance of achieving not only coverage but also an aesthetically appropriate outcome. The employment of tissue expansion techniques further enhances the reconstructive repertoire; considering factors such as defect size, trauma, and tissue distortion is vital to achieve the best result for the patient. Both our experience and the literature reviewed consider local flaps the best overall alternative for scalp defects.
